# Clinically aligned whole-body MRI segmentation of skeletal metastases via Supervised Anatomical Pretraining^☆^

**DOI:** 10.1016/j.jbo.2026.100745

**Published:** 2026-01-28

**Authors:** Joris Wuts, Jakub Ceranka, Nicolas Michoux, Frédéric Lecouvet, Jef Vandemeulebroucke

**Affiliations:** a Vrije Universiteit Brussel, Department of Electronics and Informatics, Pleinlaan 2, Brussels, 1050, Belgium; b imec, Kapeldreef 75, Leuven, B-3001, Belgium; c Cliniques universitaires Saint Luc, Institut de Recherche Expérimentale et Clinique (IREC), Université catholique de Louvain (UCLouvain), Avenue Hippocrate 10, Brussels, 1200, Belgium; d Universitair Ziekenhuis Brussel, Department of Radiology, Laarbeeklaan 101, Brussels, 1090, Belgium; e Fonds Wetenschappelijk Onderzoek (FWO), Rue de Louvain 38, Brussels, 1000, Belgium

**Keywords:** Metastatic bone disease, Whole-body MRI, Anatomical priors, Treatment response assessment, Clinical decision support

## Abstract

In oncology practice, response assessment of metastatic disease requires reliable and reproducible quantification of measurable metastatic burden. Manual identification, segmentation, and volumetry of all lesions is labor-intensive and variable, limiting routine clinical adoption. An automated approach is therefore needed. Segmenting metastatic bone disease (MBD) on whole-body MRI (WB-MRI) is challenging because of the heterogeneous appearance and anatomical distribution of lesions, ambiguous boundaries, and the low volumetric prevalence of metastatic deposits within the body. Training robust machine learning models for this task requires large, well-annotated datasets that capture lesion variability. However, assembling such datasets demands substantial expert time and is prone to annotation error. Although self-supervised learning (SSL) can take advantage of large unlabeled datasets, the learned representations tend to remain generic and may miss the subtle anatomical and pathological features essential for accurate lesion detection.

In this work, we propose a Supervised Anatomical Pretraining (SAP) method that learns from a limited dataset of anatomical labels. First, an MRI-based skeletal segmentation model is developed and trained on WB-MRI scans from healthy individuals for high-quality skeletal delineation. Then, we compare its downstream efficacy in segmenting MBD on a cohort of 40 patients with metastatic prostate cancer, against a randomly initialized baseline and a state-of-the-art self-supervised method.

SAP significantly outperforms both the Baseline and SSL-pretrained models achieving a normalized surface Dice of 0.78 and a Dice coefficient of 0.66. The method achieved a lesion detection $F_{2}$ score of 0.45, improving on 0.26 (Baseline) and 0.31 (SSL). When considering only clinically relevant lesions larger than 1 mL, SAP achieves a mean lesion level sensitivity of 0.89 at 0.46 false positives per exam, supporting reliable follow-up and treatment-response assessment.

Learning bone morphology from anatomy yields an effective and domain-relevant inductive bias that can be leveraged for the downstream segmentation task of bone lesions. These results highlight SAP’s clinical utility for standardized, high-sensitivity WB-MRI monitoring of skeletal metastases in routine bone oncology practice. All code and models are made publicly available.

## Highlights

• Supervised Anatomical pretraining (SAP) improves detection of bone metastases.
• SAP outperforms SOTA pretraining for lesion segmentation and detection.
• For lesions ≥1  mL, mean sensitivity is 0.89 at 0.46 FP/exam.
• Supports radiologist-assisted detection aligned with MET-RADS-P.
• SAP achieves lesion Dice 0.66, but global Dice 0.46 is too low for volumetry.

## Graphical abstract

**Figure:**
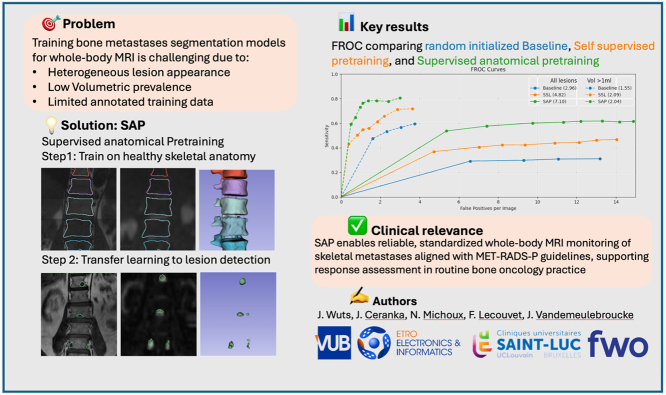

## 1 Introduction

Metastatic bone disease (MBD) is a frequent complication in advanced cancers, particularly in prostate and breast malignancies [1], [2]. In bone oncology, the clinical need is reliable, reproducible detection and quantification of lesions on serial examinations to assess response over time and guide systemic therapy. In practice, standardized guidelines such as the *Metastasis Reporting and Data System for Prostate Cancer (MET-RADS-P)* provide clear criteria for selecting and monitoring target lesions on WB-MRI [3]. Quantitative biomarkers derived from WB-MRI such as total tumor volume, diffusion volume, and the apparent diffusion coefficient (ADC) have emerged as tools for assessing disease severity and monitoring treatment response, underscoring the importance of early and accurate volumetric assessments of skeletal lesions [4], [5]. However, precise manual segmentation of these lesions remains a labor-intensive and error-prone process, motivating the development of automated and robust segmentation methods [6]. For clinical adoption, such methods must achieve high sensitivity for target lesions and maintain a very low false-positive burden to avoid unnecessary reader arbitration.

Historically, response assessment of bone metastases has been challenging. Under RECIST 1.1, most bone lesions are classified as non-measurable unless they contain a measurable soft-tissue component [7]. This limitation reflected the inadequate sensitivity of earlier imaging modalities (CT and bone scintigraphy) for quantifying marrow-replacing disease. The advent of whole-body MRI (WB-MRI), together with refined anatomic and diffusion-weighted sequences (T1-weighted, STIR, T2-Dixon, and high-b-value DWI), has filled this gap by enabling direct visualization and more precise measurement of metastatic infiltration of the bone marrow.

Importantly, early work in advanced prostate cancer demonstrated that RECIST-like quantitative assessment of bone metastases is feasible. Tombal et al. showed that MRI of the axial skeleton permits objective measurement of tumor burden and treatment response in prostate cancer bone metastases, supporting the concept that unidimensional evaluation can be extended to skeletal disease [8]. Subsequent developments in multiparametric WB-MRI, have further refined the ability to assess metastatic response reproducibly and form the basis of structured reporting systems such as MET-RADS-P [3].

Over the past decade, deep learning has become a popular method for medical image segmentation, yet its success depends on large, high-quality annotated datasets. In MBD, data collection is particularly challenging due to the sparse distribution of small lesions, inherent variability in expert annotations, and the diverse appearance of lesions in different anatomical skeletal locations. This label-scarcity problem has motivated adoption of transfer learning paradigms, where knowledge learned in one domain (e.g., large-scale natural or medical images) is applied to a related task.

Recently, self-supervised learning (SSL) has shown promise by learning general-purpose feature representations from unlabeled data through proxy tasks such as inpainting, rotation prediction, or contrastive instance discrimination [9], [10]. Despite these advances, SSL-derived representations tend to be agnostic to fine-grained anatomical details critical for delineating subtle pathological deviations. Moreover, according to an extensive recent study in medical imaging, SSL may offer marginal or even negative returns, and the influencing factors are poorly understood [11]. This observation motivates alternative strategies for pre-training networks which can serve as a robust foundation for downstream segmentation tasks. Among these, supervised pre-training on easily annotated healthy anatomy is appealing because it injects domain-relevant anatomical context that is directly useful for detecting and segmenting clinically actionable disease.

In this work, we propose a novel Supervised Anatomical Pre-training strategy and validate its performance on the task of segmentation of bone lesions from WB-MRI. Our approach leverages skeletal anatomy by first training a skeletal segmentation model on WB-MRI scans from healthy subjects and subsequently fine-tuning it for lesion segmentation within the skeleton. This strategy is inspired by the clinical observation that radiologists routinely use their understanding of normal anatomy to identify subtle anomalies within bone and marrow.

Our contributions are threefold:

• We introduce SAP, a novel supervised anatomical pre-training method designed to explicitly leverage healthy anatomy priors to improve accuracy of metastatic lesion segmentation in WB-MRI.
• We validate the effectiveness of SAP through comprehensive comparisons, demonstrating it significantly outperforms both randomly initialized models and existing state-of-the-art self-supervised methods in lesion detection and segmentation tasks in low-data regimes.
• We extensively evaluate SAP’s performance specifically in detecting and segmenting MBD in WB-MRI, underscoring its clinical utility and broader potential to enhance diagnostic accuracy and monitoring across diverse oncologic imaging scenarios. In particular, for measurable target lesions as defined by MET-RADS-P, SAP delivers high sensitivity with a low false-positive burden at clinically relevant operating points, facilitating reliable, standardized response assessment.

By explicitly harnessing healthy anatomical features, our method offers a compelling alternative to conventional transfer learning strategies, addressing both the challenges of data scarcity and the limitations of generic SSL.

## 2 Related works

In this section, we review the literature motivating our study and group prior work into two themes: MBD segmentation in clinical response assessment and lesion/skeleton segmentation, and pretraining for medical image segmentation spanning SSL and supervised approaches.

### 2.1 MBD segmentation context

Response assessment in metastatic disease is typically guided by RECIST 1.1 (for visceral organs, lymph nodes, and measurable soft-tissue disease) and, for WB-MRI in advanced prostate cancer, by MET-RADS-P. Under RECIST 1.1, purely sclerotic (blastic) bone metastases are considered non-measurable, whereas lytic or mixed bone lesions with a measurable soft-tissue component are considered measurable when the soft-tissue component has a longest diameter larger than 10 mm [7]. MET-RADS-P standardizes WB-MRI acquisition, reading, and reporting to enable reproducible, whole-skeleton response assessment in prostate cancer [3]. Inter-observer studies report that MET-RADS-P achieves good agreement across experience levels, yet reporting remains time-consuming and depends on consistent lesion selection [12]. In parallel, consensus guidance from ESR/EORTC emphasizes standardized lesion segmentation to support quantitative imaging biomarkers in trials and clinical practice [6]. Building on these frameworks, automated methods that prioritize high sensitivity for clinically actionable lesions at a low false-positive burden can accelerate routine reporting and improve trial-readiness. In this work, we adopt a pragmatic design of analyzing large target lesions (≥1mL), that roughly corresponds to a ∼12 mm spherical diameter (above MET-RADS-P 10 mm measurability) and reduces spurious detections in WB-MRI, thereby aligning algorithm performance with clinical follow-up requirements [5].

Early work by Ceranka et al. introduced an automated computer-aided diagnosis pipeline tailored for detecting and segmenting MBD from WB-MRI. By implementing a robust preprocessing pipeline, this approach surpassed prior methods, attaining an $F_{2}$-score of 0.50 for detection and a lesion segmentation Dice coefficient of 0.53. This study underscored the importance of rigorous preprocessing to enhance downstream performance, setting a benchmark for automated analysis of MBD in WB-MRI [13].

Later, Kim et al. [14] proposed a method specifically for segmenting bone metastases in spinal MRI using a multicenter dataset. Their evaluation of various combinations of MRI sequences has demonstrated superior performance with a 2D U-Net architecture integrating non-contrast $T_{1}$-weighted and contrast-enhanced fat-suppressed images. Their model achieved a sensitivity of 0.93 per lesion and a Dice coefficient of 0.70 on lesions larger than 1 ${\mathrm{cm}}^{3}$. These results confirm the potential of deep learning approaches to substantially enhance clinical evaluation of MBD, potentially streamlining radiological workflows and improving diagnostic accuracy.

Recent supervised studies on larger cohorts help place our results in a broader context for lesion detection and segmentation. Faghani et al. developed a deep learning model for detecting lytic bone lesions in multiple myeloma on CT [15], and Wennmann et al. reported multicenter MRI-based detection of focal bone marrow lesions in plasma cell disorders [16]. Although these works differ in modality and disease focus from WB-MRI metastatic prostate cancer and are not directly comparable, they underscore the gains achievable with extensive supervised data and help position our study within the wider literature.

Complementary to lesion-focused approaches, MRI skeletal and bone-marrow segmentation has advanced in recent years. Multicenter whole-body MRI studies have achieved robust bone marrow or skeletal segmentation [17], [18], and whole-body diffusion or multi-structure MRI models demonstrate broad anatomical coverage with reliable skeleton delineation [19], [20]. These efforts provide context for our skeletal pretraining stage.

### 2.2 Pretraining for medical image segmentation

Self-supervised learning has emerged as a dominant paradigm for pretraining medical imaging models without requiring extensive manual annotations. Prominent approaches include masked image modeling (e.g., Masked Autoencoder, MAE [21]), contrastive learning (e.g. SimCLR [9]), and generative tasks such as image restoration through inpainting and deformation (e.g., Model Genesis [10]). Recent benchmarks such as the OpenMIND initiative [22], systematically compared SSL methods across diverse medical tasks and imaging modalities, demonstrating that fine-grained reconstruction pretext tasks primarily benefit segmentation performance, whereas contrastive instance discrimination approaches typically enhance classification outcomes. Zhang et al. [11] provided an extensive empirical evaluation of predictive, contrastive, generative, and hybrid SSL frameworks, highlighting that no single method uniformly excels across all tasks. Their findings stress the importance of aligning SSL pretext tasks closely with downstream objectives, architectural consistency, and domain relevance, particularly emphasizing SSL’s advantage in scenarios characterized by class imbalance.

In line with these insights, Tang et al. [23] introduced a tailored SSL framework specifically for 3D medical image analysis, employing a Swin UNETR architecture that integrates masked volume inpainting, 3D rotation prediction, and contrastive learning. This combination of global context modeling and local representation learning has demonstrated state-of-the-art performance on benchmarks such as the Medical Segmentation Decathlon (MSD) [24] and Beyond the Cranial Vault (BTCV) datasets [25]. Given the dual requirement of our downstream tasks, both segmentation and detection, we adopt this SSL framework as a benchmark against our method.

Complementary to SSL approaches, supervised pretraining leveraging densely annotated datasets has consistently delivered robust performance in medical segmentation tasks. Established examples such as TotalSegmentator [26] provide extensive, voxel-wise annotations across a wide range of anatomical structures in CT images. Recently, Li et al. [27] further expanded upon these supervised pretraining paradigms through SuPreM, a unified supervised pretraining framework trained on AbdomenAtlas 1.1, comprising more than 9000 CT volumes annotated with 25 anatomical structures and additional pseudo-labels for various tumor types. Employing both CNN-based architectures (e.g., SegResNet) and transformer-based models (e.g., Swin UNETR), they illustrated how large-scale, fully annotated datasets could significantly enhance downstream segmentation tasks, particularly in scenarios with limited labeled data. Critically, supervised pretraining that encodes relevant anatomy can yield inductive biases well aligned with clinical targets, thereby improving sensitivity for larger, follow-up lesions without inflating false positives.

In contrast to the generalized pretraining approaches commonly employed in SSL frameworks such as OpenMIND and supervised methods like SuPreM, our proposed method explicitly targets MBD segmentation using WB-MRI. Generalized pretraining methods typically rely on large-scale, modality-generic datasets that predominantly encompass common imaging sequences and anatomical domains. Consequently, they often lack adaptability to specialized imaging setups such as WB-MRI, which uniquely combine modalities such as $T_{1}$-weighted and diffusion-weighted imaging (DWI) into multi-channel inputs. Adapting these generic pretrained models directly to specialized imaging modalities is challenging, primarily because datasets of adequate scale and quality to replicate the original pretraining schemes are rarely available. Our approach addresses these limitations by specifically aligning the pretraining strategy to the downstream clinical application, using a relatively small yet modality- and anatomy-specific dataset. Leveraging high-resolution anatomical representations from healthy skeletal anatomy within the same imaging domain as the downstream task allows our model to efficiently capture both global anatomical context and local structural detail essential for the simultaneous detection and segmentation of bone lesions. This targeted strategy reduces the dependence on large-scale generic datasets, enhances modality and domain consistency, and thus potentially leads to improved diagnostic accuracy and transferability.

## 3 Methodology

### 3.1 Data acquisition

We acquired two WB-MRI datasets: an anatomical dataset for supervised anatomical pretraining, and a pathology dataset to assess the downstream efficacy of the method. The anatomical dataset comprises 24 healthy volunteers [28], yielding 72 multi-parametric WB-MRI scans ($T_{1}$ and DWI, $b_{1000}{\mathrm{s/mm}}^{2}$) obtained at three different research institutes. One examination was excluded due to corrupted image quality and incomplete station coverage, leaving 71 examinations for analysis. Manual skeletal annotations were initially performed on one scan per subject using 3D Slicer [29] by trained interns who underwent training on skeletal representation in anatomical MRI sequences. These initial segmentations were subsequently propagated to the remaining scans via a piecewise linear, bone-specific registration pipeline. Final manual adjustments were then conducted by a radiology expert with seven years of experience in skeletal representations using WB-MRI.

The pathological dataset includes WB-MRI scans from 40 advanced prostate cancer patients with confirmed skeletal metastases, comprising a mixed population of hormone-sensitive metastatic prostate cancer (mHSPC) and castration-resistant metastatic prostate cancer (mCRPC). Lesions in the spine, pelvis, femurs, ribs, and clavicles were delineated according to MET-RADS-P recommendations [3]. Lesion identification followed standard WB-MRI criteria for marrow-replacing metastatic deposits (low $T_{1}$ signal, high signal on high b-value DWI ($b=1000\;{\mathrm{s/mm}}^{2}$), and abnormally increased ADC relative to adjacent normal bone marrow), consistent with MET-RADS-P response assessment practice. Manual delineations were performed in ITK-SNAP [30] by a medical imaging specialist with seven years of experience in WB-MRI and subsequently validated during a consensus session with an expert radiologist specializing in oncologic imaging and bone metastases identification using multiparametric WB-MRI.

Supplementary material (Appendix A, Table A.2 and Figure A.2) provides detailed cohort characteristics and lesion distribution summaries.

**Fig. 1:**
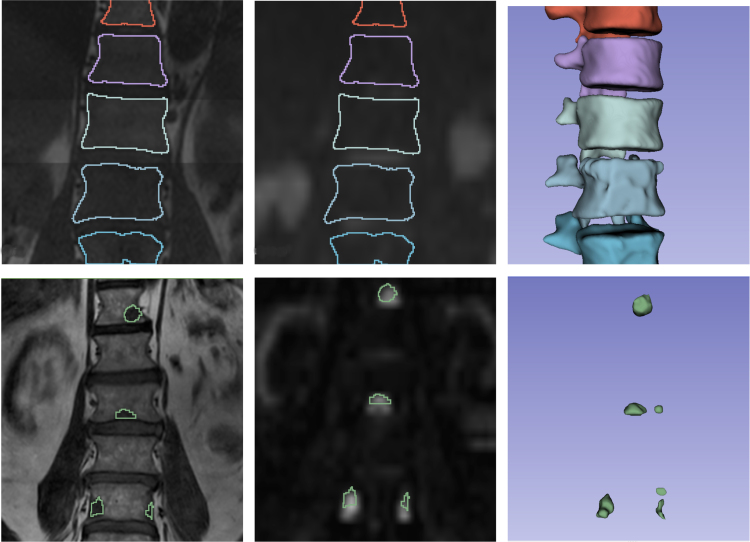
Visualization of two imaging channels and segmentation mask of coronal views of the thoracic spine: **(Left)**$T_{1}$-weighted MRI, **(Middle)**$b_{1000}$ diffusion-weighted image, and **(Right)** 3D render of the segmentation mask. The top row represents the thoracic spine of a healthy subject with a multi-label skeletal segmentation, while the bottom row depicts the thoracic and lumbar spine of a patient with metastatic bone lesions. Areas with low $T_{1}$ signal intensity together with high $b_{1000}$ signal intensity correspond to viable bone metastases.

Preprocessing, following the pipeline described in Ceranka et al. [31], involved noise and bias correction, intensity normalization, rigid registration, and whole-body stitching to form a two-channel ($T_{1}$ and DWI, $b_{1000}{\mathrm{s/mm}}^{2}$)) image stack. Fig. 1 shows the three channels ($T_{1}$, DWI, $b_{1000}{\mathrm{s/mm}}^{2}$), and segmentation mask) for a representative subject from both datasets. Detailed data acquisition and preprocessing protocols are provided in the supplementary material (Appendix A).

### 3.2 Ethics approval and consent to participate

All datasets used in this study are derived from human subjects. Processing and analysis complied with the Declaration of Helsinki, GDPR, and institutional policies.

**PICRIB healthy skeletal dataset (prospective volunteers).** Healthy volunteers were prospectively recruited for whole-body skeletal MRI at three hospitals, with Cliniques universitaires Saint-Luc (UCLouvain) serving as the coordinating (main) institute. All participants provided written informed consent. Ethics approvals (2016): UZ Jette (VUB) *2016/209*; Cliniques universitaires Saint-Luc (UCLouvain) *2016/01JUL/243*; Erasme Hospital (ULB) *P2016/273*.

**MOC-UP metastatic patient dataset (retrospective).** WB-MRI examinations from advanced prostate cancer patients were obtained from Cliniques universitaires Saint-Luc. The data were prospectively acquired under institutional ethics approval *(2019/24DEC/581)* and were retrospectively reused for this study in accordance with the approved protocol.

### 3.3 Experimental setup: metastatic bone disease

For our supervised training experiments, we employed the Swin UNETR architecture as described by Hatamizadeh et al. [32]. In our implementation, the network is configured with parameters listed in the supplementary material (Appendix B, Table B.1). The model outputs a 3D lesion mask per exam (two-channel input: $T_{1}$ and DWI, $b_{1000}{\mathrm{s/mm}}^{2}$) , which we convert into lesion candidates (connected components) for detection analysis. The task is binary: metastatic lesion vs. background. To mitigate extreme foreground–background imbalance and streamline WB-MRI training, we employ the instance-balanced patch sampler from our prior work. The sampler samples exactly one positive patch per lesion per epoch with matched negatives, improving efficiency while preserving per-lesion representation [33].

We evaluated three distinct supervised training strategies for MBD segmentation, all using the underlying Swin UNETR architecture. A summary of the three methods is given, highlighting their differences in model initialization and the subsequent fine-tuning procedure on the MBD dataset:

#### 3.3.1 Random initialization (baseline)

In the Baseline approach, the entire network is initialized with random weights and trained directly on the MBD dataset. This method serves as a reference to quantify the benefits of any pretraining strategy.

#### 3.3.2 Self supervised pretraining (SSL)

For the SSL pretrained strategy, we adopt the default self-supervised learning implementation as described in the original work of Tang et al. [23]. In this pipeline, the model is first pretrained on unlabeled WB-MRI volumes using a combination of inpainting, rotation prediction, and contrastive learning objectives. We pretrained the SSL model exclusively on the healthy volunteer WB-MRI dataset (n = 71 scans), no patient scans were used. After SSL pretraining, the encoder weights are transferred to the MBD segmentation model. The decoder is newly initialized, and the entire network is fine-tuned on the metastatic dataset.

#### 3.3.3 Supervised anatomical pretraining

In our proposed SAP approach, we leverage a previously trained model on healthy skeleton segmentation, which captures explicit bone contours in nine anatomical regions: the cervical, thoracic, and lumbar spine; pelvis; femurs; humerus; scapulae; clavicles; and sternum. For the MBD segmentation task, we transfer both the encoder and decoder weights from this model; however, the final classification head that is tailored for multi-class segmentation is discarded and replaced by a randomly initialized binary segmentation head. During fine-tuning on the metastatic dataset, we apply a differential learning rate strategy: a lower initial learning rate (e.g., $2\times 10^{-6}$) is used for the encoder to preserve the learned anatomical features, while a higher initial learning rate (e.g., $1\times 10^{-5}$) is used for the decoder to better adapt to the binary lesion segmentation task.

### 3.4 Experimental setup: Skeletal segmentation

Using the healthy volunteer dataset (24 subjects, 71 scans) described in Section 3.1, we trained a supervised skeletal segmentation model to delineate nine key anatomical skeletal regions: the cervical, thoracic, and lumbar spine; pelvis; femurs; humerus; scapulae; clavicles; and sternum, mirroring WB-MRI reading regions emphasized in routine skeletal metastases assessment. The model employs the same Swin UNETR architecture and identical training hyperparameters as used for the MBD segmentation network (supplementary material, Appendix B, Table B.1). In particular, a multi-class segmentation head with ten output channels was used to learn the aforementioned bone classes. A fixed training and validation set was made ensuring the scans originating from the same subject are within the same split. In total, we trained the model on 63 scans and validated it on the remaining 8. This approach yielded a robust anatomical model that captures healthy skeletal morphology, providing the foundation for our SAP initialization in the downstream lesion segmentation task.

### 3.5 Evaluation metrics, cross-validation, and statistical testing

Supplementary material (Appendix C, Table C.1) summarizes the evaluation metrics used in our study. We group metrics into three categories: (i) detection metrics (FPPI, lesion-level $F_{2}$-score, sensitivity, precision, FROC, and FROC-AUC), (ii) pure segmentation metrics conditioned on detection (Dice and NSD computed on correctly detected lesions), and (iii) end-to-end metrics that include missed lesions (per-patient mean Dice across lesions, with missed lesions scored as 0; global Dice computed over the whole patient with all lesions merged into a single foreground; and volume agreement using the two-way random-effects intraclass correlation coefficient (ICC(2,1), absolute agreement, single measures) [34] for total tumor burden). The evaluation metrics were selected based on the Metrics Reloaded framework [35], which also provides more detailed definitions of all metrics. During evaluation, we report detection metrics both across all lesions and specifically for lesions larger than 1mL, the latter reflecting the subset most relevant for clinical decision-making and patient follow-up in accordance to MET-RADS-P . Additionally, to assess the performance of the skeletal segmentation pretraining, we computed the segmentation Dice coefficient. In this context, we report class-specific Dice scores for each bone region, and also a global binary Dice score by merging all bone regions into a single foreground class (whole-skeleton vs. background), reflecting overall accuracy of skeleton segmentation.

Lesion burden is highly heterogeneous across patients, with many patients having only a few lesions and a small subset exhibiting extensive disease (supplementary material, Appendix A, Figure A.2). To keep lesion counts balanced across splits, we performed a stratified 7-fold cross-validation over the cohort. One fold was used as a fixed threshold-tuning split. The remaining folds formed the test splits; for each run, one fold was selected as the test split while models were trained on all other data, including the tuning split, and metrics were aggregated across the test folds. Metrics are reported on 33 test patients and do not include the tuning split. For lesion detection, we performed connected-component analysis on binarized probability maps and matched predictions to ground truth by overlap (any non-zero overlap counted as a detection); the binarization threshold was selected on the fixed tuning split to maximize the patient-level $F_{2}$ score and then applied unchanged to all test folds. For statistical testing, we first assessed normality of all metric distributions using the Shapiro–Wilk test [36], which indicated that none were normally distributed. Accordingly, comparisons between methods were performed using non-parametric tests. For patient-level (detection) and end-to-end metrics, where data are naturally paired across methods, the Wilcoxon signed-rank test [37] was used. For lesion-level (segmentation) metrics, an unpaired Wilcoxon test (Mann–Whitney U test [38]) was applied since the set of detected lesions varies between methods. Bonferroni correction [39] was applied to adjust for multiple comparisons, and p-values and the number of samples were reported where applicable.

### 3.6 Clinical relevance of the 1 mL threshold

In clinical WB-MRI practice, target lesions are selected based on MET-RADS-P measurability (on the order of 10 mm in longest diameter) to enable reproducible longitudinal tracking [3], [8]. A 1 mL spherical lesion corresponds to ∼12 mm diameter, giving a RECIST-like size proxy for measurable lesions and reducing spurious sub-mL candidates. Accordingly, we report performance across all detected lesions while emphasizing results for lesions ≥1  mL, which aligns with MET-RADS-P-focused assessment of focal target lesions. Since this study focuses on focal disease and the cohort does not include diffuse-disease cases, this threshold is appropriate here. For diffuse marrow involvement, lesion boundaries are not well defined, and thresholds based on lesion size are less meaningful.

### 3.7 Implementation details

The model was implemented using the MONAI framework [40] built on PyTorch. Training and inference were conducted on two NVIDIA Ampere GPUs with 80 GB of memory using torch Distributed Data Parallel for multi-GPU processing. All source code, including pretrained and finetuned models, are publicly available to support further research in this area on https://github.com/jwutsetro/SAP. Reproducibility details are provided in the supplementary material (Appendix A).

## 4 Results

### 4.1 Healthy skeleton segmentation

We evaluated a Swin UNETR model trained with random weight initialization on healthy WB-MRI scans to segment nine distinct bone categories. On a validation set of eight WB-MRI scans, the model achieved a mean Dice similarity coefficient of 0.90 for binary segmentation of the skeleton. Class-wise Dice scores were: cervical 0.90, thoracic 0.94, lumbar 0.93, pelvis 0.80, femur 0.90, humerus 0.67, scapula 0.70, clavicles 0.83, sternum 0.85, and full skeleton 0.90. Accurate delineation of normal skeletal morphology underpins the SAP inductive bias leveraged downstream for lesion detection/segmentation in clinically prioritized regions (spine, pelvis, femurs, clavicles, ribs).

**Fig. 2:**
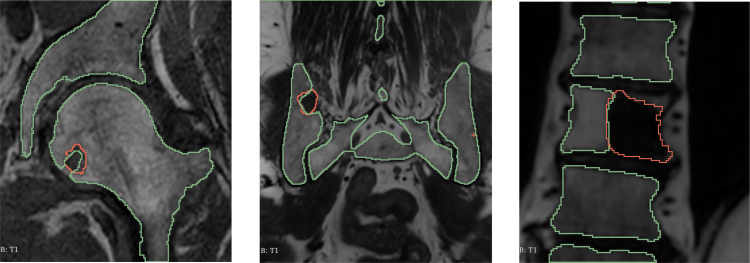
Coronal $T_{1}$-weighted views obtained from three different anatomical locations in three patients with MBD showing manual lesion segmentation (red contours) and automated skeletal predictions (green contours). From left to right, the images display a femur with one metastatic lesion, a pelvis with one lesion, and a thoracic vertebra with one lesion.

Fig. 2 displays three coronal $T_{1}$-weighted images from patients with MBD overlaid with the skeleton prediction maps. These examples demonstrate that while the model robustly delineates most healthy bone structures, its performance deteriorates in regions affected by metastatic infiltration. Specifically, the model systematically fails to include bone tissue affected by metastases in the overall skeletal segmentation, and healthy tissue adjacent to lesions is not accurately delineated. This behavior is clinically plausible: pathologic marrow replacement alters $T_{1}$/DWI, $b_{1000}{\mathrm{s/mm}}^{2}$ appearance and can degrade purely anatomical segmentation near lesions.

### 4.2 Initial feature representation prior to finetuning

**Fig. 3:**
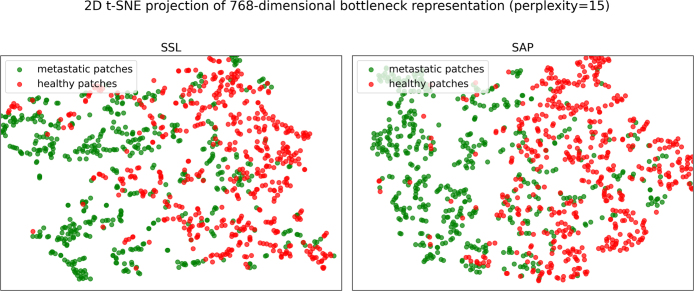
Two-dimensional t-SNE projections of the 768-dimensional bottleneck features (perplexity = 15) learned via two different pretraining strategies prior to fine-tuning on a MBD dataset. The left panel shows the distribution of patches learned under SSL, and the right panel shows the distribution from SAP. Green points correspond to patches labeled as positive for MBD, whereas red points correspond to negative patches.

We visualized the feature representations derived from the SSL and SAP models using a 2D t-SNE projection to capture the global structure of the 768-dimensional bottleneck features of the Swin UNETR. These features were extracted from the models prior to any finetuning on the MBD dataset, thereby reflecting the initialized models’ inherent capability to discriminate between lesions and healthy patches. For both methods, all lesion patches and a randomly selected equal number of healthy patches were processed by the models. As shown in Fig. 3, the SAP method exhibits a better class separation compared to the SSL method. The corresponding cluster metrics computed on the full feature space reveal similar inter-cluster (centroid) distances for the two methods (SSL: 9.85, SAP: 9.85). However, the intra-cluster distances differ. For SSL, the average intra-cluster distances are 21.31 for positives and 21.37 for negatives, whereas for SAP they are 10.02 for positives and 10.44 for negatives. Both models are thus positioning the centroids of the positive and negative clusters at comparable distances, however the features from the SAP model are substantially more compact. Together, the t-SNE projection plot and cluster metrics illustrate that before finetuning, the initialized SAP model is more effective at discriminating positives from negatives compared to the SSL model. This pre-finetuning separation anticipates the downstream gains observed on clinically actionable lesions.

### 4.3 Metastatic bone disease segmentation

For the MBD segmentation task, we compared three training methods as described in section 3.2. All models were fine-tuned on the metastatic cohort and evaluated using stratified 7-fold cross-validation with one fixed threshold-tuning split; performance is aggregated across the six test folds (33 test patients).

#### 4.3.1 Quantitative evaluation metrics

**Fig. 4:**
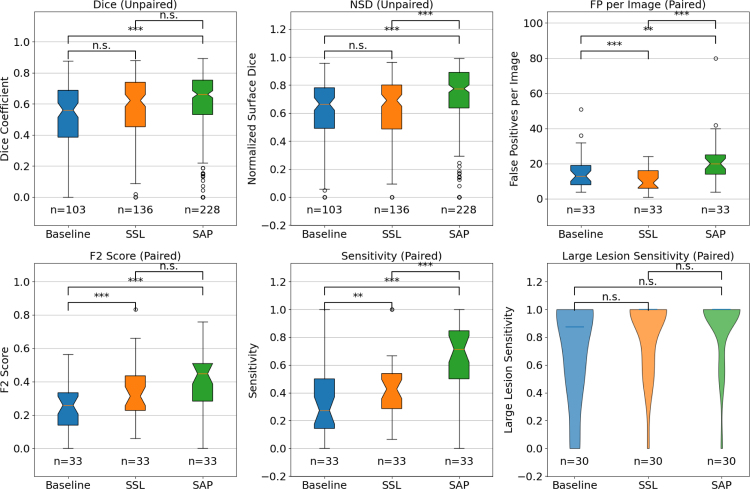
Comparison of detection and segmentation metrics for MBD across three methods (Baseline, SSL, and SAP). Metrics include Dice Coefficient, normalized surface Dice (NSD), false positives per exam, $F_{2}$-Score, detection sensitivity, and large lesion detection sensitivity. Statistical significance is denoted by * (p<0.05), ** (p<0.01), and *** (p<0.001), with ’n.s.’ indicating no significance. For the segmentation metrics, the number of detected lesions per method that were included in the distribution is indicated with n. For the detection metrics, n indicates the number of patients included in the distribution. Large lesion sensitivity is computed only for patients with at least one lesion ≥1 mL.

Fig. 4 presents box plots summarizing detection and segmentation metrics for the Baseline, SSL, and SAP methods, including statistical significance. The obtained results reveal a consecutive progression in detection and segmentation performance: the SSL model improves upon the Baseline, and the SAP approach further improves upon the SSL method. Worth noting is that the SAP model consistently outperforms both the Baseline and SSL-pretrained models in the segmentation metrics (e.g., Dice, Normalized Surface Dice) and detection sensitivity.

Detection metrics are reported first. The median sensitivity increases from 0.27 for the Baseline to 0.43 for SSL, and reaching 0.71 with SAP, while the $F_{2}$-score improves from 0.26 (Baseline) to 0.31 (SSL) and 0.45 (SAP). Precision remains modest at this recall-optimized operating point (Baseline 0.14, SSL 0.20, SAP 0.17). Although the SAP model exhibits a higher false positive rate (20.0 FP per exam) compared to the Baseline (13.0) and SSL (9.0), this increase in FPPI comes with improved sensitivity and overall detection quality. FROC curves and FROC-AUC values are reported separately in Section 4.4.

Pure segmentation metrics conditioned on detection also improved: Dice scores increased from 0.56 (Baseline) to 0.62 (SSL) and 0.66 (SAP), and NSD improved from 0.67 to 0.69 and 0.78, respectively. End-to-end metrics that include missed lesions showed similar gains: the per-patient mean Dice increased from 0.13 (Baseline) to 0.24 (SSL) and 0.44 (SAP) [Baseline vs. SSL p<0.001; Baseline vs. SAP p<0.001; SSL vs. SAP p<0.001], and global Dice (lesion vs. background over the whole patient) improved from 0.21 to 0.33 and 0.46 [SAP vs. SSL p<0.001; SAP vs. Baseline p<0.001; SSL vs. Baseline p<0.001]. ICC(2,1) agreement for total tumor burden was 0.309 for Baseline, 0.608 for SSL, and 0.649 for SAP. See Table 1 for a comprehensive overview of the exact median values and interquartile ranges.

For clinical interpretation, we additionally summarize performance restricted to lesions ≥1mL. At this operating point (indicated on Fig. 6), median large-lesion sensitivity increases from 0.88 (Baseline) to 1.00 (SSL and SAP), supporting reliable follow-up of target lesions.

**Table 1:** Detection and segmentation metrics for Baseline, SSL, and SAP. The median with 95% confidence intervals (estimated via nonparametric bootstrapping with 1000 resamples) and the 25%–75% quartiles are shown.

Metric	Method	Median [95% CI]	25%–75% Quartiles
*Detection (all lesions)*			
Sensitivity	Baseline	0.273 [0.250–0.455]	0.143–0.500
	SSL	0.427 [0.333–0.500]	0.286–0.538
	SAP	0.714 [0.533–0.786]	0.500–0.846
Precision	Baseline	0.143 [0.111–0.200]	0.071–0.214
	SSL	0.200 [0.125–0.300]	0.120–0.400
	SAP	0.167 [0.143–0.201]	0.121–0.261
FP per exam	Baseline	13.000 [8.000–16.000]	8.000–19.000
	SSL	9.000 [7.000–14.000]	6.000–16.000
	SAP	20.000 [17.000–24.000]	14.000–25.000
$F_{2}$ Score	Baseline	0.259 [0.152–0.286]	0.139–0.333
	SSL	0.312 [0.274–0.408]	0.227–0.435
	SAP	0.448 [0.357–0.484]	0.283–0.508
*Detection (lesions*≥1mL*)*			
Sensitivity	Baseline	0.875 [0.583–1.000]	0.500–1.000
	SSL	1.000 [0.792–1.000]	0.667–1.000
	SAP	1.000 [1.000–1.000]	1.000–1.000
FP per exam	Baseline	0.000 [0.000–1.000]	0.000–1.000
	SSL	0.000 [0.000–0.000]	0.000–0.000
	SAP	0.000 [0.000–0.000]	0.000–1.000

*Segmentation (detected lesions)*			
Dice	Baseline	0.560 [0.512–0.615]	0.385–0.687
	SSL	0.622 [0.563–0.649]	0.453–0.739
	SAP	0.662 [0.629–0.687]	0.531–0.753
NSD	Baseline	0.665 [0.613–0.702]	0.491–0.781
	SSL	0.691 [0.621–0.727]	0.487–0.801
	SAP	0.775 [0.747–0.820]	0.637–0.891

*End-to-end*			
Dice (per-patient mean)	Baseline	0.131 [0.093–0.185]	0.033–0.249
	SSL	0.241 [0.163–0.264]	0.126–0.293
	SAP	0.442 [0.351–0.525]	0.255–0.548
Global Dice	Baseline	0.212 [0.162–0.345]	0.049–0.453
	SSL	0.333 [0.201–0.483]	0.147–0.571
	SAP	0.463 [0.318–0.606]	0.271–0.635

#### 4.3.2 FROC analysis

**Fig. 5:**
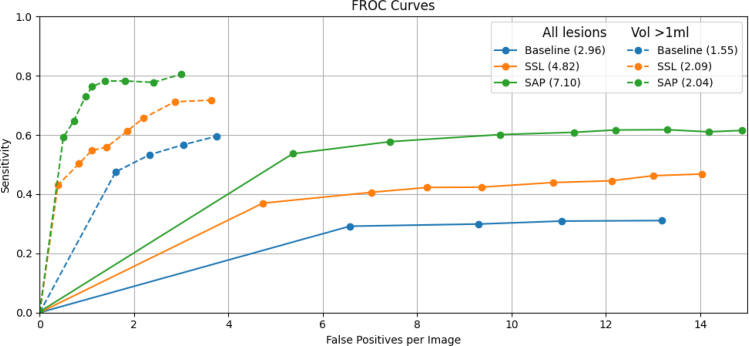
Combined FROC curves for lesion detection: solid lines show all lesions up to 15 FPPI, and dashed lines show lesions with volume ≥ 1 mL up to 4 FPPI, for the Baseline, SSL, and SAP methods. The FROC-AUC is numerically integrated under each piecewise-linear curve to its respective FPPI limit and reported in the legend.

Fig. 5 illustrates the FROC curves for the three methods, plotting lesion detection sensitivity against false positives per exam (up to 15 FPPI). A stepwise improvement is observed: the Baseline method performs worse than the SSL approach, which in turn is outperformed by the SAP model. This trend is confirmed by the higher FROC AUC values (2.96, 4.82 and 7.10 respectively), with SAP achieving the greatest area under the curve. Moreover, the curves remain nearly parallel, demonstrating that for any given FPPI threshold, sensitivity increases progressively from Baseline to SSL and from SSL to SAP. We observe similar trends when restricting the analysis to clinically relevant lesions larger than 1 mL (dashed lines). All three methods achieve higher sensitivities and fewer false positives per exam in this setting, reflecting the improved detectability of larger lesions. The relative ranking of the methods remains unchanged, with SAP consistently outperforming both SSL and Baseline approaches across the full range of FPPI values.

#### 4.3.3 Sensitivity and lesion volume analysis

**Fig. 6:**
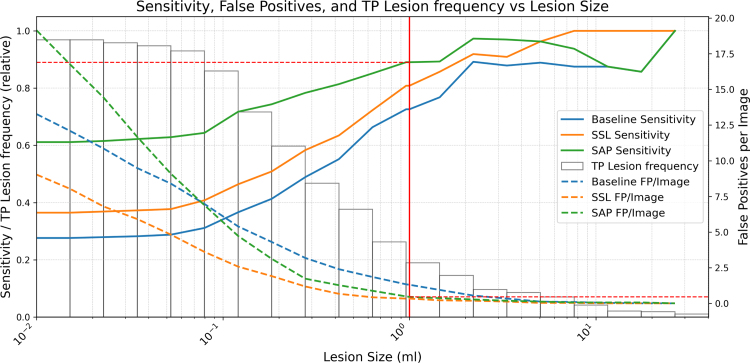
Combined evaluation of sensitivity and false positives per exam (FP/exam) for three methods (Baseline, SSL, SAP) as a function of lesion size. Sensitivity (solid lines) and FP/exam (dashed lines) are plotted against lesion volume; the left y-axis corresponds to sensitivity and the right y-axis to FP/exam. Both metrics are cumulative, considering all lesions larger than the given volume. The white histogram represents the cumulative distribution of true lesion volumes, providing insight into lesion volume prevalence. The vertical red line marks the clinically emphasized operating point at ≥1 mL (approximately 12 mm spherical diameter), used throughout the manuscript and aligned with MET-RADS-P measurability.

To evaluate the influence of lesion size on detection performance, we analyzed sensitivity and FPPI as a function of lesion volume (Fig. 6). All methods exhibit reduced sensitivity for small lesions; however, for lesions ≥1 mL (clinically relevant), sensitivity improves stepwise: from 0.73 for the Baseline, to 0.81 for SSL, and up to 0.89 for SAP. Mean FPPI concurrently drops to 1.30, 0.33, and 0.46, respectively. These ≥1 mL lesions account for only 25% of all annotations, and most false positives arise from sub-mL candidates, underscoring the value of an explicit minimum prediction volume. Clinically, restricting analysis to lesions ≥1 mL yields a manageable arbitration burden and supports standardized follow-up: SAP reaches 0.89 lesion-level sensitivity at 0.46 FP/exam, compared with 0.81/0.33 for SSL and 0.73/1.30 for the Baseline.

#### 4.3.4 Qualitative visualization

**Fig. 7:**
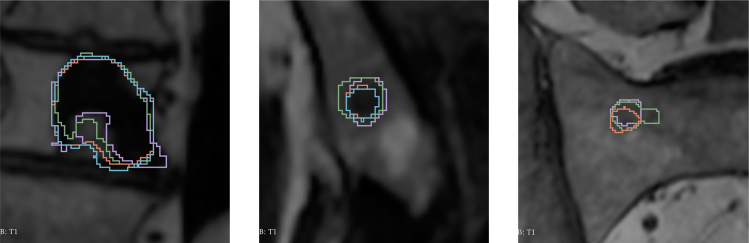
Coronal $T_{1}$-weighted MR images from subjects with metastatic lesions are shown with manual annotations in purple. Overlaid are the predicted segmentations from three training paradigms: Baseline (blue), SSL (orange), and SAP (green). The panels (left to right) depict lesions in the cervical spine, right iliac bone, and sacrum, selected to represent the 25th, 50th, and 75th percentiles of Dice scores (based on the SAP model), respectively. In the cervical lesion, all methods detect the lesion but tend to oversegment the inferior concave margin, with segmentation quality improving from Baseline to SSL to SAP. In the iliac lesion, SAP slightly oversegments the lesion whereas Baseline and SSL undersegment it. In the sacral lesion, the Baseline model fails to detect the lesion, while SSL and SAP capture it with residual undersegmentation and oversegmentation, respectively, illustrating the variability in segmentation performance across methods and lesion difficulty.

Qualitative inspection of the model outputs shows that both pretraining strategies reduce detection failures and improve the delineation of metastatic lesions compared with the randomly initialized baseline. In particular, the pretrained models yield segmentations that more closely follow lesion boundaries at bone–marrow interfaces and along concave margins, although residual oversegmentation and undersegmentation remain for anatomically complex sites. These observations are consistent with the quantitative gains in Dice, normalized surface Dice, and lesion-level sensitivity reported above and are illustrated on representative cases in Fig. 7.

#### 4.3.5 Results on metastatic bone disease segmentation

Our experimental results indicate that the Baseline method is not able to capture the complex morphology of skeletal lesions, resulting in lower segmentation accuracy and detection performance. The SSL approach, offers moderate improvements by leveraging unlabeled data, but does not incorporate the anatomical priors necessary for precise lesion delineation. The SAP strategy significantly outperforms the other methods by achieving higher segmentation accuracy and improved lesion detection metrics. Crucially for bone oncology workflows, SAP sustains high sensitivity for clinically actionable lesions at a low False positive burden.

## 5 Discussion

We propose a supervised anatomical pretraining strategy for WB-MRI that learns skeletal morphology on healthy scans and transfers this inductive bias to MBD segmentation. Across the six test folds (7-fold CV with a fixed threshold-tuning split), SAP improved both detection and delineation compared with random initialization and a strong self-supervised learning baseline. Median sensitivity increased stepwise (Baseline 0.27, SSL 0.43, SAP 0.71) with concomitant gains in Dice (0.56, 0.62, 0.66) and normalized surface Dice (0.67, 0.69, 0.78). When analyses were restricted to measurable target-sized lesions (volume ≥1 mL), SAP achieved a mean lesion-level sensitivity of 0.89 at 0.46 mean false positives per exam.

### 5.1 Positioning with prior work and why anatomical pretraining helps

#### Skeletal segmentation as a foundation.

Our healthy-anatomy model reached a mean binary skeleton Dice of 0.90 on validation, with strong performance across vertebral levels, pelvis, and femora, and expected reductions in thinner bones (scapula/humerus). This provides high-fidelity context at bone–marrow interfaces where metastases perturb $T_{1}$/DWI, $b_{1000}{\mathrm{s/mm}}^{2}$ appearance; Fig. 2 shows that skeletal delineation can fail in the immediate neighborhood of lesions, suggesting the model inherently also has a form of representation for lesions. Prior WB-MRI skeleton/bone-marrow segmentation studies report Dice values in the 0.76–0.89 range depending on input modality and label granularity (e.g., Wennmann et al. Bauer et al. Candito et al. Ceranka et al.); these differences in targets, anatomy coverage, and acquisition resolution limit direct numeric comparison [17], [18], [19], [41]. Our model is multi-label, trained on higher-resolution WB-MRI (1.2 mm out-of-plane), and covers a broader set of structures than many prior evaluations (including scapulae, clavicles, and sternum). Because small, thin structures have high surface-to-volume ratios, even minor boundary offsets can cause a pronounced drop in Dice; this sensitivity to thin anatomy is a known benchmarking limitation and explains the lower scores observed for those regions [42]. Direct numeric comparisons remain limited by differences in segmentation targets, anatomy coverage, and acquisition resolution, but the overall performance supports the model as a foundation for transfer learning in downstream metastatic lesion segmentation. High-resolution anatomical pretraining is especially important when transferring to downstream tasks that also operate at high resolution, where smaller lesions benefit from finer spatial detail.

#### SAP vs. SSL.

Before any MBD fine-tuning, SAP produced more compact, class-separable feature clusters than SSL (average intra-cluster distances ∼10 for SAP vs. ∼21 for SSL), consistent with a stronger structure-aware inductive bias. In practice, this translated into a consistently higher FROC curve and the largest FROC-AUC among methods. Notably, SAP achieved these gains with markedly lower pretraining budget (5.5 GPU-hours) than the SSL pipeline (101 GPU-hours).

#### Comparison with disease-specific literature and scope of benchmarking.

Recent supervised studies on metastatic bone disease have used substantially larger annotated cohorts, including lytic lesion detection on CT by Faghani et al. achieving sensitivity 91.6%, specificity 84.6%, and AUROC 0.904 [15], and focal bone marrow lesion detection on MRI by Wennmann et al. with sensitivity 0.49/0.34 and PPV 0.61/0.61 on internal/external test sets (mAP 0.44/0.34) [16]. These works target different modalities, disease contexts, lesion definitions, and evaluation protocols, which limits direct numeric comparison to our WB-MRI setting. On spinal MRI, prior work reported per-lesion sensitivity and Dice up to 0.93/0.70 for lesions ≥1 cm${}^{3}$. Our WB-MRI task spans spine *and* pelvis/femora/clavicles with a greater fraction of sub-mL lesions; within that broader and harder setting, SAP delivered a mean large-lesion sensitivity of 0.89 for lesions ≥1 mL and competitive Dice on detected lesions. Because modality, cohort definition, lesion criteria, and 2D/3D setups vary across the literature, we focus our direct comparison on SAP versus SSL and random initialization to assess the impact of anatomical pretraining as a technique rather than an end-to-end system.

#### Data regime and pretraining impact.

We operate in a low data and label regime where both SSL and SAP improve downstream performance relative to training from scratch. We expect pretraining to remain beneficial as datasets grow, consistent with large-scale anatomy-aware pretraining efforts such as SuPreM [27] and SSL in 3D medical imaging (Models Genesis [10]). At the same time, as labeled datasets expand, marginal gains from pretraining generally diminish, so the relative advantage of SAP/SSL may narrow even though absolute performance improves.

### 5.2 Volume agreement, end-to-end evaluation, and total tumor burden estimates

Agreement between reference and predicted total tumor burden ranged from fair (Baseline ICC(2,1) 0.309) to good (SSL ICC(2,1) 0.608; SAP ICC(2,1) 0.649). End-to-end segmentation metrics that include missed lesions were modest but improved with pretraining: the per-patient mean Dice increased from 0.13 (baseline) to 0.24 (SSL) and 0.44 (SAP). This is expected in a setting dominated by many small lesions and a small number of larger targets: missed small lesions contribute zero volume, while partial over- or under-segmentation of a few larger lesions disproportionately affects patient-level volume. These findings indicate that although SAP improves these metrics, it is not yet suitable for absolute whole-body burden estimation.

### 5.3 Clinical utility and workflow integration

The operating characteristics of SAP align with how bone oncologists read WB-MRI under MET-RADS-P for focal target lesions. Three points stand out.

1. **Radiologist detection assistance.** For lesions ≥1 mL, SAP reaches a mean lesion-level sensitivity of 0.89 at 0.46 FP/exam, supporting candidate triage in standardized reporting such as MET-RADS-P.
2. **Initial segmentation for volumetry.** The delineation quality on detected lesions (Dice 0.66; NSD 0.78) provides reasonable starting contours that can be refined for volumetry, allowing consistent tracking of ADC and volume changes over time.
3. **Total tumor burden.** Modest end-to-end Dice and only fair-to-good volume agreement indicate the current system is not yet a complete whole-body tumor-burden estimator.

### 5.4 Error profile and interpretability

False positives were concentrated among small (<1  mL) candidates and interpretable skeletal changes such as healed fractures or treatment-related sclerosis. Because SAP “reasons through” anatomy first, these errors tend to be understandable imaging findings rather than random artefacts. We also observed cases where the model’s smooth boundary better matched hypointense $T_{1}$ regions than the manual contour, underscoring the inherent variability of hand-drawn GT delineations.

### 5.5 Limitations

This is a single-center study with a modest healthy cohort (24 volunteers) and 40 patients, all with advanced prostate cancer, so broader generalizability across vendors, protocols, and primary tumors remains to be shown. The patient cohort is inherently male-only and drawn from a single hospital, limiting demographic diversity and potentially under-representing variation in body compositions and disease phenotype. Although the healthy dataset spans three sites, it remains small and does not capture the full variability across scanner vendors, field strengths, coil configurations, and protocol tuning that can affect WB-MRI intensity distributions and DWI/ADC measurements. We relied on internal cross-validation without a fully independent external test set; therefore, performance may be optimistic relative to true multicenter deployment across heterogeneous institutions. Ground-truth quality constrained evaluation: (i) some detected lesions lay outside the original annotation field and were penalized as false positives; and (ii) inter-observer variability at indistinct margins likely affected per-lesion Dice. Finally, many lesions were small (median 0.5 mL), where sensitivity is intrinsically lower and FP rates are less clinically relevant but influence global metrics.

### 5.6 Future directions

We plan a multicenter, multi-vendor prospective reader study that deploys SAP as a target-lesion assistant embedded in a MET-RADS-P workflow. Primary endpoints will include time-to-report, inter-reader agreement for lesion selection, lesion-level sensitivity at fixed FP/exam (0.2 and 0.5), and concordance of AI-assisted response categories with clinical outcomes.

### 5.7 Summary

Supervised anatomical pretraining delivers clinically aligned improvements in WB-MRI detection and segmentation of skeletal metastases, with high sensitivity and a low candidate burden for measurable lesions. These properties map naturally onto MET-RADS-P reporting and trial workflows and argue for prospective evaluation as a second-reader safety net and target-lesion assistant in routine bone oncology practice.

## 6 Conclusion

Supervised anatomical pretraining leverages healthy skeletal morphology on WB-MRI to provide a strong, domain-relevant inductive bias for metastatic bone disease segmentation. In the six test folds (7-fold CV with a fixed threshold-tuning split), SAP consistently outperformed both random initialization and a strong self-supervised baseline, improving lesion detection and delineation (Dice 0.66; NSD 0.78).

Critically for bone oncology workflows, SAP attains high performance on clinically actionable disease. For target-sized lesions (volume ≥1 mL), SAP achieved a lesion-level sensitivity of 0.89 at 0.46 false-positive candidates per exam, a workload compatible with routine MET-RADS-P reporting and longitudinal follow-up. These properties support two immediate use cases: (i) target-lesion assistance to accelerate selection and volumetry; and (ii) a second-reader safety net to flag new or enlarging targets with minimal arbitration overhead.

Because SAP “reasons through” anatomy first, typical false positives (e.g., healed fractures, treatment-related sclerosis) are interpretable findings rather than random artefacts, facilitating safer integration into structured reports. While demonstrated in a single-center prostate cancer cohort, the concept generalizes to settings where learning normal anatomy confers advantage.

All source code, pretrained weights, and study models are publicly available at https://github.com/jwutsetro/SAP to support reproducibility and further clinical validation.

## CRediT authorship contribution statement

**Joris Wuts:** Writing – original draft, Visualization, Validation, Software, Methodology, Investigation, Funding acquisition, Formal analysis, Conceptualization. **Jakub Ceranka:** Writing – review & editing, Supervision, Data curation. **Nicolas Michoux:** Writing – review & editing, Resources, Data curation. **Frédéric Lecouvet:** Writing – review & editing, Supervision, Resources, Funding acquisition, Data curation. **Jef Vandemeulebroucke:** Writing – review & editing, Supervision, Project administration, Funding acquisition.

## Consent for publication

No individual person’s identifiable data are included in this article. Consent for publication is therefore not applicable.

## Declaration of Generative AI and AI-assisted technologies in the writing process

During the preparation of this work, the author(s) used ChatGPT (GPT-5, OpenAI) to assist with formatting tables and figures and to improve academic clarity. After using this tool, the author(s) reviewed and edited all content and take full responsibility for the content of the published article.

## Declaration of competing interest

The authors declare that they have no known competing financial interests or personal relationships that could have appeared to influence the work reported in this paper.

## Data Availability

The MRI datasets analyzed in this study contain patient data and are not publicly available due to GDPR and institutional privacy regulations. Source code, pretrained weights, and trained study models are available at https://github.com/jwutsetro/SAP.

## Appendix A Supplementary data

MMC S1 Detailed data acquisition and preprocessing protocols for the healthy and advanced prostate cancer WB-MRI datasets. MMC S1

MMC S2

MMC S3
