# Supplementary material for: Clinically aligned whole-body MRI segmentation of skeletal metastases via Supervised Anatomical Pretraining
Source: J Bone Oncol. 2026 Jan 28;57:100745. doi: 10.1016/j.jbo.2026.100745 (PMC12890717; doi:10.1016/j.jbo.2026.100745)
Supplement: MMC S1 — Detailed data acquisition and preprocessing protocols for the healthy and advanced prostate cancer WB-MRI datasets. [file mmc1.pdf]

Supplementary File: Appendix A – Data Acquisition and Preprocessing

A.1. Data Acquisition and Preprocessing

In what follows, we detail both the healthy dataset from the Platform for Imaging in Clinical Research in Brussels study (PICRIB) and the pathological dataset from advanced prostate cancer patients.

A.1.1. Healthy Dataset: The PICRIB Study and Skeletal Segmentation

Originally, the PICRIB study [1] was designed to assess the repeatability and reproducibility of ADC measurements in a multicenter WB-MRI protocol. In this work, this standardized dataset is repurposed to support high-quality skeletal segmentation and anatomical pretraining. The healthy cohort consisted of 24 asymptomatic volunteers (10 women and 14 men, aged 23–57 years) recruited from three academic centers in Belgium: Cliniques Universitaires Saint Luc (Université catholique de Louvain), UZ Brussel (Vrije Universiteit Brussel), and Hôpital Erasme, Brussels (Université libre de Bruxelles). Volunteers underwent two scans at one institute and an additional scan at another institute to evaluate both intra- and inter-center reproducibility. All examinations were performed using the same 3.0-T MRI scanner model (Philips Ingenia 3.0 T).

Manual Skeleton Labeling For each subject in the healthy dataset, manual skeletal labeling was performed on one 3D T_1-weighted scan using 3D Slicer [2]. Initial annotations were generated by trained operators following standardized guidelines, with approximately one subject's labeling completed per workday. These annotations were subsequently refined by a medical imaging expert with over 7 years of experience in bone anatomy to ensure precise delineation of cortical boundaries.

Semi-Automated Piecewise Bone Registration Pipeline To propagate the skeletal annotations across multiple scans per subject, a semi-automated piecewise bone registration pipeline was implemented. The registration protocol consisted of 3 steps:

1. **Global Rigid Registration:** Each subject's annotated scan was first globally aligned to the target scan to achieve a rough overall correspondence.
2. **Localized Bone-Specific Registration:** For each bone, a 10-voxel dilated mask was generated to isolate the region of interest. A localized rigid registration was then applied to precisely align the masked regions.
3. **Sequential Spinal Registration:** Special attention was given to the spinal column. Registration was performed sequentially, starting at the sacrum, with each vertebra registered in turn. The transformation from the preceding vertebra was used as the initialization for the next, ensuring robust alignment even for the smaller vertebral bodies.

The proposed piecewise strategy effectively accommodated inter-scanner variability in pixel intensities and subtle anatomical deformations arising from differences in patient positioning and surrounding soft tissue. An example of the refined skeletal annotations can be seen in Figure A.1.

Registration Parameters The registration steps were implemented using SimpleElastix [3] with the following parameters: the multi resolution registration framework was employed with an Euler-transform and a B-Spline interpolator for registration. The optimizer was adaptive stochastic gradient descent, configured with a maximum of 2000 iterations and 3 resolution levels. Advanced Mattes Mutual Information similarity metric was used. Additional settings included a 10-voxel dilation of the bone masks, an image pyramid schedule of (4,4,2,2,2,1,1,1,1), with 2048 spatial samples per iteration.

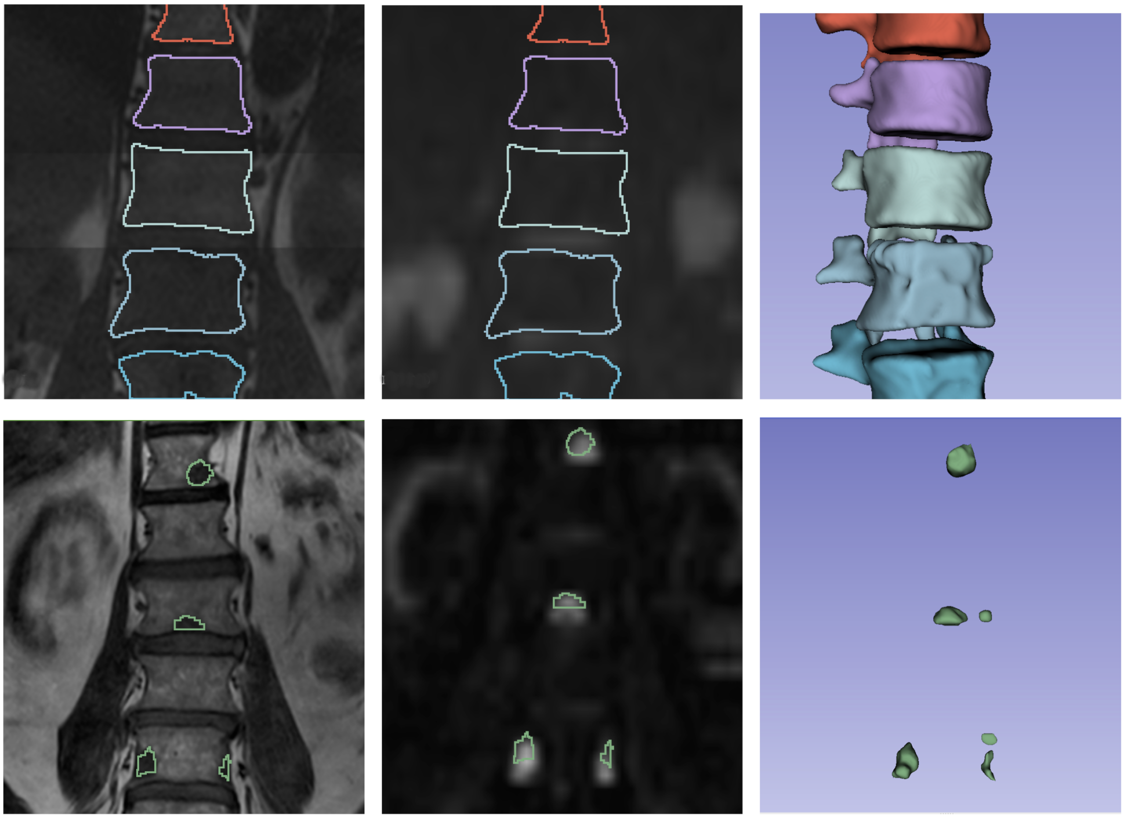

Figure A.1: Examples of refined multi-class skeletal segmentation. **Left:** 3D render of the included skeletal bones. **Right:** A sagittal T_1-weighted view on the spinal column from a healthy subject with annotated skeleton.

A.1.2. Pathological Dataset: Multi-Parametric WB-MRI in Advanced Prostate Cancer

The pathological dataset consists of WB-MRI scans acquired from 40 advanced prostate cancer patients with confirmed skeletal metastases during routine clinical examinations at Cliniques Universitaires Saint-Luc, Brussels, Belgium. The imaging protocol combined anatomical and functional sequences, namely:

- **Anatomical Sequences:** Acquisitions using either a 3D T_1-weighted or an in-phase DIXON sequence [4].
- **Diffusion-Weighted Imaging (DWI):** Images were acquired at multiple b-values (0, 50, 150, and 1000 s/mm^2), with the high b-value (b_{1000}) images being critical for visual lesion detection in this study [5].

Manual Lesion Segmentation Metastatic lesions in the whole spine, pelvis, femurs, and clavicles were manually delineated on high-resolution T_1-weighted images following MET-RADS-P criteria [6]. The manual segmentation was performed using ITK-SNAP and/or 3D Slicer [2, 7] as the primary software tool. Initial annotations were carried out by trained researchers and subsequently refined by a medical imaging specialist with over 7 years of experience in oncology imaging. A total of 340 lesions larger than 50 voxels were annotated with this approach. Lesion burden distributions across patients are summarized in Figure A.2.

Acquisition Parameters Table A.1 summarizes the key acquisition parameters for the three primary imaging sequences used in the pathological dataset. Cohort characteristics are summarized in Table A.2.

Table A.1
Acquisition parameters for the MRI sequences in the pathology dataset.

Parameter	3DTSE T_1	3DGRE T_1 DIXON	DWI, $b_{1000}s/mm^2$)
TE (ms)	8	1.15	66
TR (ms)	382	3.6	8421
Matrix Size	480×480	432×432	192×192
Pixel Spacing (mm)	0.65	1.04	2.3
Slice Thickness (mm)	1.1	1.5	6.1

Table A.2
Cohort and dataset characteristics for the healthy and pathological WB-MRI datasets.

Characteristic	Healthy (PICRIB)	Pathological (MOC-UP)
Age, mean (range)	F: 38 (25–54); M: 37 (23–57)	69.51 (36–85)
Sex, n (%)	F: 10 (41.7%); M: 14 (58.3%)	M: 40 (100%)
Subjects (n)	24	40
Scans (n)	71 (1 excluded)	40
Field strength / vendor / model	3.0 T Philips Ingenia	3.0 T Philips Ingenia
Sequence types (n)	2 (WB 3D T1-weighted; WB-DWI $b = 0/150/1000\,s/mm^2$)	2 (3D T1-weighted or in-phase DIXON; DWI $b = 0/50/150/1000\,s/mm^2$)

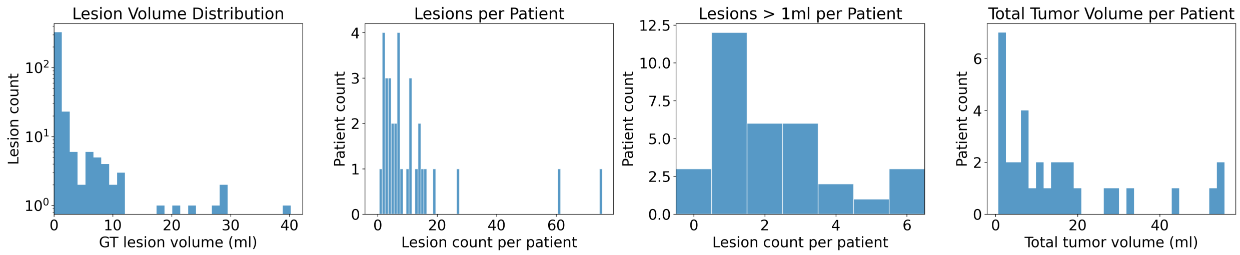

Figure A.2: Lesion burden histograms in the pathological cohort. Distributions are shown for lesion volume, lesions per patient, lesions >1 mL per patient, and total tumour volume. The pathological cohort includes 340 total lesions, of which 84 are ≥ 1 mL.

A.1.3. Comprehensive Preprocessing Pipeline for Whole-Body MRI

Both datasets were processed using a robust preprocessing framework designed to address the inherent challenges of WB-MRI, such as spatial distortions and intensity inhomogeneities [8]. The pipeline comprises the following key steps:

1. Noise Suppression and Bias Field Correction Anisotropic diffusion filtering [9] was applied to reduce noise while preserving critical edge information, followed by the N4ITK non-parametric non-uniform intensity normalization algorithm to correct for low-frequency bias fields [10].

2. Inter-Station Intensity Standardization A linear intensity matching was performed in the overlapping regions of adjacent image stations (Inter-Station Intensity Standardization, as described in the work of Ceranka et al. [8]) to ensure consistent intensity profiles across the different stations.

3. Spatial Registration of DWI Stations Rigid registration was employed to align sequential diffusion-weighted imaging stations using the pelvis as the reference, thereby correcting for inter-station misalignments Ceranka et al. [11].

4. Whole-Body Image Reconstruction and Resampling The registered stations were stitched together into a continuous whole-body volume via linear interpolation in overlapping areas, ensuring smooth transitions. Subsequently, all DWI images were resampled to match the resolution of the anatomical sequences.

5. Inter-Modality Image Registration Following the reconstruction of whole-body volumes, DWI images were registered to the anatomical (TSE or GRE T_1) images using a rigid registration approach supplemented by deformable adjustments as necessary. This step guarantees accurate spatial correspondence between modalities.

6. Inter-Patient Intensity Standardization A piecewise linear scaling algorithm was applied to standardize intensities across patients. Intensity histograms were aligned based on predetermined percentiles (0, 20, 40, 60, 80, and 95) to mitigate inter-subject variability [12].

The integrated preprocessing pipeline has been demonstrated to significantly enhance the performance of our computer-aided diagnosis (CAD) system in prior work of Ceranka et al. [8].

References

[1] N. F. Michoux, J. W. Ceranka, J. Vandemeulebroucke, F. Peeters, P. Lu, J. Absil, P. Triqueneaux, Y. Liu, L. Collette, I. Willekens, C. Brussaard, O. Debeir, S. Hahn, H. Raeymaekers, J. de Mey, T. Metens, F. E. Lecouvet, Repeatability and reproducibility of ADC measurements: a prospective multicenter whole-body-MRI study, Eur. Radiol. 31 (2021) 4514–4527.

[2] A. Fedorov, R. Beichel, J. Kalpathy-Cramer, J. Finet, J.-C. Fillion-Robin, S. Pujol, C. Bauer, D. Jennings, F. M. Fennessy, M. Sonka, J. Buatti, S. R. Aylward, J. V. Miller, S. Pieper, R. Kikinis, 3d slicer as an image computing platform for the quantitative imaging network, Magnetic Resonance Imaging 30 (2012) 1323–1341.

[3] K. Marstal, F. Berendsen, M. Staring, S. Klein, Simpleelastix: A user-friendly, multi-lingual library for medical image registration, in: Proceedings of the IEEE Conference on Computer Vision and Pattern Recognition Workshops (CVPRW), 2016, pp. 574–582. doi:10.1109/CVPRW.2016.78.

[4] V. Pasoglou, N. Michoux, F. Peeters, A. Larbi, B. Tombal, T. Selleslagh, P. Omoumi, B. C. Vande Berg, F. E. Lecouvet, Whole-body 3d t1-weighted mr imaging in patients with prostate cancer: Feasibility and evaluation in screening for metastatic disease, Radiology 275 (2015) 155–166. Published online 2014-12-15.

[5] T. Takahara, Y. Imai, T. Yamashita, S. Yasuda, S. Nasu, M. Van Cauteren, Diffusion weighted whole body imaging with background body signal suppression (dwibs): technical improvement using free breathing, stir and high resolution 3d display, Radiation Medicine 22 (2004) 275–282.

[6] A. R. Padhani, F. E. Lecouvet, N. Tunariu, D.-M. Koh, F. De Keyzer, D. J. Collins, E. Sala, H. P. Schlemmer, G. Petralia, H. A. Vargas, S. Fanti, H. B. Tombal, J. de Bono, METastasis reporting and data system for prostate cancer: Practical guidelines for acquisition, interpretation, and reporting of whole-body magnetic resonance imaging-based evaluations of multiorgan involvement in advanced prostate cancer, Eur. Urol. 71 (2017) 81–92.

[7] P. A. Yushkevich, Y. Gao, G. Gerig, ITK-SNAP: An interactive tool for semi-automatic segmentation of multi-modality biomedical images, in: 2016 38th Annual International Conference of the IEEE Engineering in Medicine and Biology Society (EMBC), 2016, pp. 3342–3345.

[8] J. Ceranka, J. Wuts, O. Chiabai, F. Lecouvet, J. Vandemeulebroucke, Computer-aided diagnosis of skeletal metastases in multi-parametric whole-body MRI, Comput. Methods Programs Biomed. 242 (2023) 107811.

[9] P. Perona, T. Shiota, J. Malik, Anisotropic diffusion, in: B. M. ter Haar Romeny (Ed.), Geometry-Driven Diffusion in Computer Vision, Springer Netherlands, Dordrecht, 1994, pp. 73–92. URL: https://doi.org/10.1007/978-94-017-1699-4_3. doi:10.1007/978-94-017-1699-4_3.

[10] N. J. Tustison, B. B. Avants, P. A. Cook, Y. Zheng, A. Egan, P. A. Yushkevich, J. C. Gee, N4itk: improved n3 bias correction, IEEE Transactions on Medical Imaging 29 (2010) 1310–1320. Epub 2010 Apr 8.

[11] J. Ceranka, M. Polfliet, F. Lecouvet, N. Michoux, J. de Mey, J. Vandemeulebroucke, Registration strategies for multi-modal whole-body MRI mosaicing, Magn. Reson. Med. 79 (2018) 1684–1695.

[12] J. Ceranka, F. Lecouvet, N. Michoux, J. de Mey, H. Raeymaekers, T. Metens, J. Vandemeulebroucke, Comparison of intra- and inter-patient intensity standardization methods for multi-parametric whole-body mri, Biomed Phys Eng Express 9 (2023). Epub 2023 Apr 10.
