# Supplementary material for: Clinically aligned whole-body MRI segmentation of skeletal metastases via Supervised Anatomical Pretraining
Source: J Bone Oncol. 2026 Jan 28;57:100745. doi: 10.1016/j.jbo.2026.100745 (PMC12890717; doi:10.1016/j.jbo.2026.100745)
Supplement: MMC S2 [file mmc2.pdf]

Supplementary File: Appendix B – Training Configuration

Table B.1: Supervised training model architecture parameters. The model architecture is derived from the work of Hatamizadeh et al. [1].

Component	Details
**Input**	Patch size: $96 \times 96 \times 96$ voxels; In channels: 2; Out channels: 2
**Encoder**	• Feature Size: 48<br>• Encoder Depths: [2, 2, 6, 2]<br>• Window Size: 7^3<br>• Patch Size: 2^3<br>• Drop Rate: 0.1; Attention Drop Rate: 0.1; Drop Path Rate: 0.1
**Decoder**	Standard U-Net style decoder using transposed convolutions and skip connections.
**Optimizer & LR**	AdamW with a base learning rate of 1×10^{-5} and weight decay of 1×10^{-5}
**Scheduler**	Cosine annealing learning rate scheduler with a linear warmup phase (warmup epochs = 40, total epochs = 400)
**Data Augmentation**	Random crop, random flips, intensity shifts.

References

[1] A. Hatamizadeh, V. Nath, Y. Tang, D. Yang, H. R. Roth, D. Xu, Swin unetr: Swin transformers for semantic segmentation of brain tumors in mri images, in: A. Crimi, S. Bakas (Eds.), Brainlesion: Glioma, Multiple Sclerosis, Stroke and Traumatic Brain Injuries (BrainLes 2021), volume 12962 of *Lecture Notes in Computer Science*, Springer, Cham, 2022, pp. 272–284. doi:10.1007/978-3-031-08999-2_22.
