# Supplementary material for: Clinically aligned whole-body MRI segmentation of skeletal metastases via Supervised Anatomical Pretraining
Source: J Bone Oncol. 2026 Jan 28;57:100745. doi: 10.1016/j.jbo.2026.100745 (PMC12890717; doi:10.1016/j.jbo.2026.100745)
Supplement: MMC S3 [file mmc3.pdf]

# Supplementary File: Appendix C – Evaluation Metrics

Table C.1: Overview of evaluation metrics. The selection was guided by the Metrics Reloaded work Maier-Hein et al. [1].

| Metric | Description |
|---|---|
| **Detection** | |
| FPPI (False Positives per exam) | Average number of false positive detections per exam. Reported for all lesions and for lesions $\geq$ 1 mL. |
| Lesion-Level $F_2$-Score | Combination of precision and recall with extra emphasis on recall. |
| Sensitivity | Proportion of true lesions that are correctly detected. Reported for all lesions and for lesions $\geq 1mL$. |
| Precision | Proportion of detected lesions that are true lesions. |
| FROC | Free-response Receiver Operator curve (computed up to 15 FPPI). |
| FROC-AUC | Area under the FROC curve up to the specified FPPI limit. |
| **Segmentation (detected lesions)** | |
| Dice Similarity Coefficient (DSC) | Quantifies volumetric overlap between predicted and ground-truth segmentations (computed on correctly detected lesions). |
| Normalized Surface Dice (NSD) | Measures the agreement of segmentation boundaries with a 2-voxel tolerance to account for annotation uncertainty. |
| **End-to-end** | |
| Dice (per-patient mean) | Mean of per-lesion Dice within each patient, with missed lesions scored as 0. |
| Global Dice (patient-level) | Dice computed over the whole patient volume with all lesions merged into a single foreground (lesion vs. background). |
| Volume agreement (ICC(2,1)) | Intraclass correlation coefficient (ICC(2,1), two-way random, absolute agreement, single measures) between predicted and reference total tumor burden across patients. |

## References


[1] L. Maier-Hein, A. Reinke, P. Godau, M. D. Tizabi, F. Buettner, et al. Metrics reloaded: recommendations for image analysis validation, Nat. Methods 21 (2024) 195–212.
